# Landscape genomics to the rescue of a tropical bee threatened by habitat loss and climate change

**DOI:** 10.1111/eva.12794

**Published:** 2019-04-10

**Authors:** Rodolfo Jaffé, Jamille C. Veiga, Nathaniel S. Pope, Éder C. M. Lanes, Carolina S. Carvalho, Ronnie Alves, Sónia C. S. Andrade, Maria C. Arias, Vanessa Bonatti, Airton T. Carvalho, Marina S. de Castro, Felipe A. L. Contrera, Tiago M. Francoy, Breno M. Freitas, Tereza C. Giannini, Michael Hrncir, Celso F. Martins, Guilherme Oliveira, Antonio M. Saraiva, Bruno A. Souza, Vera L. Imperatriz‐Fonseca

**Affiliations:** ^1^ Instituto Tecnológico Vale Belém Brazil; ^2^ Departamento de Ecologia Universidade de São Paulo São Paulo Brazil; ^3^ Departamento de Biociências Universidade Federal Rural do Semi‐Árido Mossoró Brazil; ^4^ Instituto de Ciências Biológicas Universidade Federal do Pará Belém Brazil; ^5^ Department of Integrative Biology University of Texas Austin Texas; ^6^ Departamento de Genética e Biologia Evolutiva Universidade de São Paulo São Paulo Brazil; ^7^ Departamento de Genética, Faculdade de Medicina de Ribeirão Preto Universidade de São Paulo Ribeirão Preto Brazil; ^8^ Unidade Acadêmica de Serra Talhada Universidade Federal Rural de Pernambuco Serra Talhada Brazil; ^9^ Centro de Agroecologia Rio Seco Universidade Estadual de Feira de Santana Amélia Rodrigues Brazil; ^10^ Departamento de Zootecnia Universidade Federal do Ceará Fortaleza Brazil; ^11^ Departamento de Sistemática e Ecologia Universidade Federal da Paraíba João Pessoa Brazil; ^12^ Escola Politécnica da Universidade de São Paulo Universidade de São Paulo São Paulo Brazil; ^13^ Embrapa Meio‐Norte Teresina Brazil

**Keywords:** deforestation, environmental associations, gene flow, isolation by resistance, local adaptation, pollination, single nucleotide polymorphism, stingless bees

## Abstract

Habitat degradation and climate change are currently threatening wild pollinators, compromising their ability to provide pollination services to wild and cultivated plants. Landscape genomics offers powerful tools to assess the influence of landscape modifications on genetic diversity and functional connectivity, and to identify adaptations to local environmental conditions that could facilitate future bee survival. Here, we assessed range‐wide patterns of genetic structure, genetic diversity, gene flow, and local adaptation in the stingless bee *Melipona subnitida,* a tropical pollinator of key biological and economic importance inhabiting one of the driest and hottest regions of South America. Our results reveal four genetic clusters across the species’ full distribution range. All populations were found to be under a mutation–drift equilibrium, and genetic diversity was not influenced by the amount of reminiscent natural habitats. However, genetic relatedness was spatially autocorrelated and isolation by landscape resistance explained range‐wide relatedness patterns better than isolation by geographic distance, contradicting earlier findings for stingless bees. Specifically, gene flow was enhanced by increased thermal stability, higher forest cover, lower elevations, and less corrugated terrains. Finally, we detected genomic signatures of adaptation to temperature, precipitation, and forest cover, spatially distributed in latitudinal and altitudinal patterns. Taken together, our findings shed important light on the life history of *M. subnitida* and highlight the role of regions with large thermal fluctuations, deforested areas, and mountain ranges as dispersal barriers. Conservation actions such as restricting long‐distance colony transportation, preserving local adaptations, and improving the connectivity between highlands and lowlands are likely to assure future pollination services.

## 1 INTRODUCTION

Although bees are now widely acknowledged as key pollinators of wild and cultivated plants, as well as important income sources for beekeepers around the globe (Potts et al., 2016), the joint impact of habitat degradation and climate change is currently threatening their wild populations (Brown & Paxton, 2009; Hadley & Betts, 2011; Potts et al., 2010; Viana et al., 2012; Wratten, Gillespie, Decourtye, Mader, & Desneux, 2012). Habitat loss has been related to reductions in native bee abundance and richness (Kennedy et al., 2013), whereas land use changes have fragmented populations in some species (Jha, 2015; Jha & Kremen, 2013). Additionally, climate change is expected to modify the availability of floral and nesting resources and affect bee physiology, thereby resulting in distribution range shifts and reductions in many species (Faleiro, Nemésio, & Loyola, 2018; Giannini et al., 2017; Kerr et al., 2015; Le Conte & Navajas, 2008; Pyke, Thomson, Inouye, & Miller, 2016; Willmer, 2014).

Landscape genomics offers powerful tools to assess the influence of habitat loss on genetic diversity and functional connectivity, and to identify adaptations to local environmental conditions that could facilitate future bee survival (Balkenhol et al., 2017; Lozier & Zayed, 2017). For instance, landscape resistance to gene flow has been assessed in both temperate and tropical species (Davis, Murray, Fitzpatrick, Brown, & Paxton, 2010; Jackson et al., 2018; Jaffé, Castilla, et al., 2016), and genomic signatures of adaptations to environmental conditions have been identified in the honeybee *Apis mellifera* (Chávez‐Galarza et al., 2013; Henriques et al., 2018) and the bumblebee *Bombus lapidarius* (Theodorou et al., 2018). Nonetheless, while most studies assessing landscape effects on gene flow have employed microsatellite markers (Balkenhol, Cushman, Waits, & Storfer, 2016; Monteiro et al., 2019), none has thus far employed genomic data to assess both isolation by landscape resistance and local adaptation in bees (Storfer, Patton, & Fraik, 2018).

Dispersal is believed to be particularly restricted in stingless bees (Apidae: Meliponini), because daughter colonies rely on resources from their maternal colonies during their initial establishment and consequently do not establish far from each other (Roubik, 2006; Van Veen & Sommeijer, 2000; Vit, Pedro, & Roubik, 2013). As restricted dispersal implies a diminished ability to move to high‐quality habitats and maintain gene flow across fragmented landscapes, habitat loss and fragmentation are expected to reduce and isolate stingless bee populations, making them extremely susceptible to genetic erosion through the action of genetic drift (Allendorf, Luikart, & Aitken, 2013; Lozier & Zayed, 2017). However, previous studies using microsatellite markers were not able to detect an effect of forest or land cover on stingless bee gene flow, suggesting that these bees have a remarkable ability to maintain high gene flow across heterogeneous and human‐altered landscapes (Jaffé, Castilla, et al., 2016; Jaffé, Pope, et al., 2016; Landaverde‐González et al., 2017). Since estimates of genetic diversity and gene flow are strongly influenced by the type and number of genetic markers employed (Allendorf, 2017; Leroy et al., 2018; Lozier, 2014), genomic studies employing thousands of single nucleotide polymorphisms (SNPs) are needed to confirm whether stingless bees are really resilient to habitat loss and fragmentation, or whether the lack of significant isolation‐by‐resistance effects in previous studies is due to the resolution of the genetic markers employed (Alvarado‐Serrano, Van Etten, Chang, & Baucom, 2019; McCartney‐Melstad, Vu, & Shaffer, 2018).

Here, we employ novel landscape genomic tools to assess the joint influence of habitat degradation and climate change on an economically important tropical stingless bee inhabiting one of the most deforested, driest, and hottest regions of the Americas. Distributed across northeastern Brazil, *Melipona subnitida* Ducke, 1911 (known as the Jandaíra bee) is a key pollinator of native plants and local crops and one of the most widely used stingless bee species for honey production (Imperatriz‐Fonseca, Koedam, & Hrncir, 2017), contributing to the household income of many rural families (Giannini et al., 2017; Jaffé et al., 2015). The species natural range spans four different biomes (Giannini et al., 2017; Imperatriz‐Fonseca et al., 2017), namely Tropical Dry Forest (Caatinga), Savanna (Cerrado), the Atlantic Rain Forest, and Mangrove Forests, thus encompassing important climatic and altitudinal gradients, which are expected to drive local adaptations (Koffler et al., 2015; Maia‐Silva, Hrncir, Silva, & Imperatriz‐Fonseca, 2015). Species distribution models suggest the species could respond to climate change by seeking refuge in higher elevations, where both the bees and their plant resources are more likely to find suitable climatic conditions in the future (Giannini et al., 2017). However, such response depends on the bee's ability to relocate to high‐quality habitats, which could be hindered by the region's increasing human‐led desertification (Marengo, Torres, & Alves, 2017; Vieira et al., 2015). Given *M. subnitida*'s key biological and economic importance, efforts are urgently needed to safeguard this key pollinator by facilitating its migration toward higher lands. Such conservation actions will nevertheless require the prior identification of barriers to gene flow and the spatial distribution of adaptive genetic variation.

Relying on thousands of SNPs, we explicitly tested whether genetic diversity and gene flow in *M. subnitida* are affected by the amount of reminiscent natural habitats, and identified genomic signatures of adaptations to local environmental conditions. Considering the life history characteristics of our study species and the high statistical power granted by the large number of genetic markers employed, we formulated the following predictions: (a) Since natural habitats harbor floral resources and nesting sites (Vit et al., 2013), we expected to find a positive association between genetic diversity and the amount of reminiscent natural habitats surrounding sampling sites (DiLeo & Wagner, 2016; Fahrig, 2013), as found in bumblebees (Jackson et al., 2018); (b) as restricted dispersal implies reduced gene flow across deforested areas, we expected to find significant isolation by landscape resistance (McRae, 2006) and predicted that gene flow would be influenced by habitat amount as well as environmental correlates of genetic connectivity in other bee species, including elevation, terrain roughness, temperature, and precipitation (El‐Niweiri & Moritz, 2011; Jackson et al., 2018; Jaffé, Pope, et al., 2016; Jha, 2015); and (c) based on the documented tolerance of our study species to extreme heat and water scarcity (Maia‐Silva et al., 2015), and previous candidate genes found associated with precipitation and latitude in honeybees (Chávez‐Galarza et al., 2013; Henriques et al., 2018) and urban land cover in bumblebees (Theodorou et al., 2018), we expected to find genomic signatures of adaptation related to temperature, precipitation, and forest cover. Ours constitutes the first genomic study assessing both isolation by landscape resistance and local adaptation across the full distribution range of a bee pollinator.

## 2 MATERIALS AND METHODS

### 2.1 Sampling and DNA extraction

**Figure 1:**
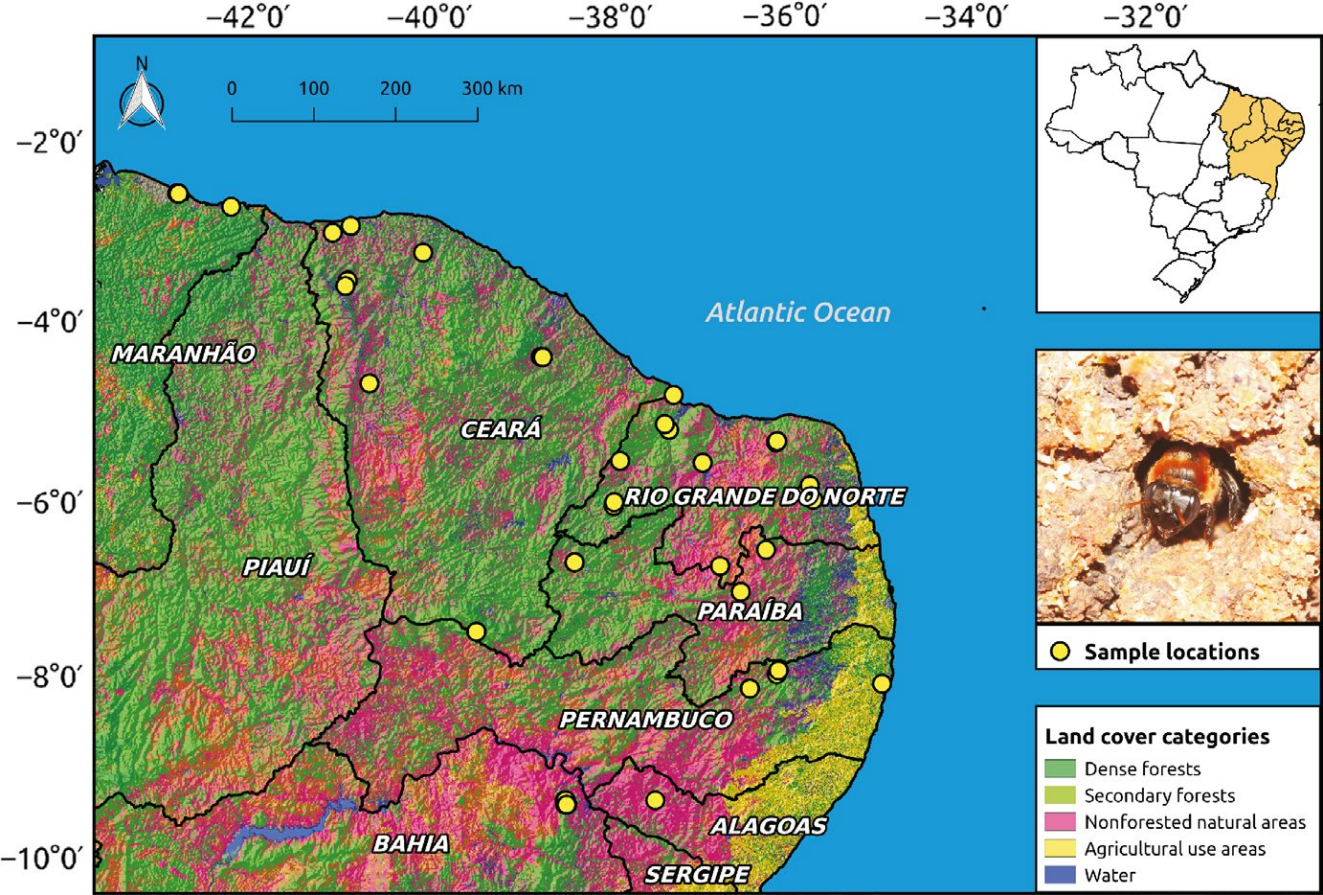
*Melipona subnitida* sampling locations across northeastern Brazil over a land cover map (source: http://mapbiomas.org/)

We collected samples of *M. subnitida* across its entire distribution range (Pedro, 2014), aiming to maximize temperature, precipitation, elevation, and forest cover gradients (Figure 1). Samples were collected between 2013 and 2014 (SISBIO collection permits 38000‐1 and 10393‐1). We sampled one bee per colony from a total of 160 nests of beekeepers who could certify their local origin. We only collected samples from local colonies, and not from colonies of unknown origin or from nests brought from different locations. We also registered any information on previous introductions of bee colonies that beekeepers could provide, as long‐distance colony transportation is common and has been shown to have profound effects on stingless bee gene flow (Jaffé, Pope, et al., 2016). In cases when beekeepers had a few weak colonies, we did not collect any samples or collected samples from a single colony. When beekeepers had many strong colonies, we collected samples from more than one colony. Distance separating sampling locations therefore ranged between 0 km (samples from the same beekeeper) and 947 km. Only freshly emerged (callow) workers were collected from the nest's interior, to minimize the sampling of drifters. All individuals were stored in absolute ethanol and then frozen at −20°C until DNA extractions. The geographic location of all samples was recorded by GPS (see Supporting Information Table S1).

Total genomic DNA was extracted from the whole insects (discarding only the heads) using Qiagen's DNeasy Blood and Tissue Kit, according to the manufacturer's protocols. DNA integrity was then verified on 1% agarose gels, and its concentration was quantified with a Qubit 2.0 Fluorometer (Invitrogen). Only samples containing nondegraded DNA, concentrations of at least 20 ng/μl, and a total DNA amount greater than 2 μg were used for subsequent RAD sequencing.

### 2.2 RAD sequencing and SNP discovery

DNA samples were then shipped to Floragenex, Inc. (Eugene, OR, USA) for RAD library preparation, Illumina sequencing, and bioinformatic processing. Briefly, libraries were prepared based on the genome size of this species (Tavares, Carvalho, Aparecida, & Soares, 2010), digesting genomic DNA with the *SgrAI* restriction enzyme. The resulting fragments were tagged with individual barcodes, which were then multiplexed and sequenced using 100‐bp single‐end methodology on the HiSeq 2000 platform (Illumina). Libraries were created at the same time and ran on the same sequencing run on two different lanes. Total number of generated reads per individual ranged from ~550,000 to 2.3 million. Samples were then demultiplexed and barcode sequences trimmed to result in final fragment lengths of 92 bp. Quality filtering was done in SAMtools during the genotyping stages, and coverage was limited to loci having less than 500× to limit the incidence of possible contaminants. Stacks was then used to cluster loci from a single individual and generate a RAD reference, allowing two haplotypes from each locus. The de novo clustering of sequences into RAD tags was performed using VELVET (version 1.2.10), considering a minimum cluster depth of 5 and maximum of 1,500, a maximum number of two haplotypes per cluster, and a maximum of three variants per cluster. SNP calling was performed using SAMtools (version 0.1.16), and loci harboring SNPs were included in the final genotype (VCF) table if they had at least 6× individual sequence coverage over at least 75% of the population, individual per locus genotype quality scores of at least 10, a minimum FASTQ quality score of 20, and a minimum distance to other SNPs of 50 bp (average individual Phred score was 60.8, while average individual sequencing coverage was 36.3×). A variant was cataloged when it was present in a single sample (only 3% of loci had minor allele frequencies—MAF—below 0.5, 2% of loci had MAF below 0.3, and 1% of loci had MAF below 0.1.).

### 2.3 Population structure and genetic diversity

The R package *r2vcftools* (https://github.com/nspope/r2vcftools), a wrapper for VCFtools (Danecek et al., 2011), was used to perform final filtering and quality control on the genotype data. To assess genetic diversity and population structure across the distribution range of our study species*,* we first filtered loci for quality (Phred score 30–80), read depth (20–50), linkage disequilibrium (LD, *r* ^2^ < 0.4), and strong deviations from the Hardy–Weinberg equilibrium (HWE, *p* < 0.0001). Additionally, we removed any potential loci under selection detected through genome scans. *F*_ST_ outlier tests were applied after adjusting *p*‐values using the genomic inflation factor (*λ*), and setting false discovery rates to *q* = 0.05, using the Benjamini–Hochberg algorithm (Benjamini & Hochberg, 1995; François, Martins, Caye, & Schoville, 2016).

Two complementary genetic clustering software packages were employed to assess population structure using the resulting set of neutral and independent loci: the *snmf* function of the LEA (v2.0) package (Frichot & François, 2015; Frichot, Mathieu, Trouillon, Bouchard, & François, 2014) and Admixture (Alexander, Novembre, & Lange, 2009). The number ancestral populations (*k*) was allowed to vary between 1 and 10, with 10 replicate runs for each *k*‐value, and the best *k* was chosen based on cross‐entropy and cross‐validation errors (Frichot et al., 2014). Individuals were then assigned to genetic clusters based on the ancestry coefficients retrieved from LEA (Q‐matrix), identifying the cluster with the highest ancestry.

We then calculated genetic diversity metrics for each one of the identified genetic clusters. These included observed heterozygosity (*H*_O_), expected heterozygosity (*H*_E_), nucleotide diversity (*π*), and inbreeding coefficient (*F*; see VCFtools manual for details). Effective population size (*N*_e_) was also estimated employing the linkage disequilibrium method implemented in NeEstimator 2.0.1 (Do et al., 2014), using a threshold lowest allele frequency value of 0.05 and assuming a monogamy model (Jaffé et al., 2014). Additionally, we calculated Tajima's *D*, representing the difference between the mean number of pairwise differences and the number of segregating sites. In a population of constant size evolving under mutation–drift equilibrium, Tajima's *D* is expected to be zero; negative values result from an excess of rare alleles and thus indicate a recent selective sweep or a population expansion after a recent bottleneck; and positive values appear when rare alleles are lacking, therefore suggesting balancing selection or a sudden population contraction (Tajima, 1989). We used *r2vcftools* to compute a genome‐wide estimate of Tajima's *D* and perform a simulation from the neutral model to correct for bias due to a minor‐allele‐frequency filter.

### 2.4 Landscape genetic analyses

Aiming to assess the influence of habitat amount on genetic diversity, we reclassified a high‐resolution land cover/land use map for 2013 (http://mapbiomas.org/) into habitat (i.e., all types of natural forest and nonforest formations) and nonhabitat (farming, nonvegetated areas, and water bodies). The percentage of habitat cover in 2‐km‐radius buffers surrounding our sampling locations was then calculated, as such radius comprises the estimated foraging distance for this species (Silva & Ramalho, 2016). Because more than one colony was sampled in some locations (Supporting Information Table S1), we computed mean percentage of habitat cover and mean genetic diversity (*H*_O_, *H*_E_, and *F*) for each location (*N* = 56 locations). We then used the *nlme* package (Pinheiro, Bates, DebRoy, & Sarkar, 2018) to fit generalized least squares models (gls) containing genetic diversity metrics as response variables, percentage of habitat cover as predictor, and different correlation structures (no autocorrelation, linear, exponential, Gaussian, spherical, and rational quadratics) to account for spatial autocorrelation. Logit transformations were used to normalize/linearize heterozygosities. The sample‐size‐corrected Akaike information criterion (AICc) was then used to compare models with different correlation structures, fitted with restricted maximum likelihood. The best models (∆AICc ≤ 2) were finally selected, fitted once more using maximum likelihood, and compared to reduced models without predictor variables using likelihood ratio tests (LRT, *α* = 0.05). All models were validated by plotting residual versus fitted values and by checking for residual autocorrelation.

Prior to assessing isolation by landscape resistance (IBR), we evaluated fine‐scale spatial genetic structure by quantifying spatial autocorrelation in genetic relatedness. To do so, we used local polynomial fitting (LOESS) of pairwise relatedness to pairwise geographic distance (https://github.com/rojaff/Lplot; Bruno, Macchiavelli, & Balzarini, 2008). Yang's relatedness between pairs of individuals (Yang et al., 2010) was used, since similar measures of relatedness have been found to be highly accurate as individual‐based genetic distance metrics for landscape genetic studies (Shirk, Landguth, & Cushman, 2017). We then evaluated the contribution of habitat amount, elevation, terrain roughness, temperature, and precipitation in explaining patterns of gene flow (Balkenhol et al., 2016). We created a first resistance surface using the reclassified land cover/land use map for 2013 described above, attributing low resistance (0.1) to habitat pixels and high resistance (0.9) to nonhabitat pixels. This allowed us to test whether gene flow is enhanced by natural habitats or hindered by habitat‐deployed environments (Jaffé, Castilla, et al., 2016). A second resistance surface was then created using an inverted forest cover map from the University of Maryland (http://earthenginepartners.appspot.com/science-2013-global-forest/download.html), to test for higher gene flow across forested areas. To test for a reduced gene flow across highlands and corrugated terrains, we created elevation and terrain roughness surfaces, using raw elevation and terrain roughness maps. While elevation was retrieved from WorldClim (http://www.worldclim.org/), terrain roughness was created from this elevation layer using the Terrain Analysis plug‐in in QGIS V2.14. To evaluate resistance due to environmental conditions, we created three additional resistance surfaces containing the raw values from those WorldClim bioclimatic variables explaining most variation across our study region (mean temperature of coldest quarter, temperature annual range, and precipitation of driest quarter; see details below). We thereby tested for reduced gene flow across areas with higher temperatures, higher temperature range, and higher precipitation. To assess isolation by geographic distance (IBD), we created a last resistance surface replacing all pixel values in our elevation map with 0.5. Using the program Circuitscape V4.0 (McRae, 2006), we then calculated pairwise resistance distances between all samples, employing all the resistance surfaces described above. Due to Circuitscape's computing limitations, all rasters were cropped to the extent of sample locations plus a buffer area of one decimal degree to minimize border effects (Jaffé, Castilla, et al., 2016), and all pixels containing zero values were replaced with 0.001.

To assess IBR, we fit mixed‐effects regression models using penalized least squares and a novel correlation structure designed to account for the nonindependence of pairwise distances across individuals and spatial locations (based on the maximum‐likelihood population effects or MLPE model: https://github.com/nspope/corMLPE; Clarke, Rothery, & Raybould, 2002). Because more than one colony was sampled in some locations (Supporting Information Table S1), there exists a spatial dependence structure in pairwise comparisons between individuals that is not captured by the MLPE model. This is because the MLPE correlation structure only models dependence across pairwise comparisons that overlap in the individuals being compared, with no reference to spatial location. The unmodeled spatial dependence has the net effect of making inference anticonservative and can be diagnosed by calculating serial autocorrelation in the normalized residuals of the MLPE model, after sorting the data by spatial locations. To combat this, we introduce a novel modification of the MLPE model that incorporates correlation between pairwise measurements due to comparison of both individuals and spatial locations (Nested MLPE or NMLPE). Briefly, the original MLPE model has a random‐effects representation where the expected value for each pairwise observation includes a pair of iid random effects (one for each of the individuals being compared in the observation), whereas our extension (NMLPE) additionally incorporates iid random effects for pairs of spatial locations (see Supporting Information Script S1 and Data S1).

Yang's relatedness between pairs of individuals was used as response variable and the different resistance distances (geographic distance, forest cover, elevation, roughness, temperature annual range, mean temperature of coldest quarter, and precipitation of driest quarter) as predictors in our MLPE models. We used the Akaike information criterion (AIC) to compare models containing all possible combinations of noncollinear predictors (*r* < 0.6), created with the *dredge* function from the MuMIn (v1.4) package (https://github.com/rojaff/dredge_mc; Barton, 2018). Likelihood ratio tests were then performed to assess the influence of the inclusion of each predictor variable on the best‐fitting model's log‐likelihood. Finally, we refitted the best models using NMLPE models to obtain parameter estimates unbiased by spatial dependence. To evaluate the impact of excluding samples from our IBR analyses, we also ran a sensitivity analysis, generating one hundred data subsets (randomly excluding different numbers of samples) and performing one hundred independent model selection protocols for each subset (using MLPE models). We report the number of times predictor variables were included in the set of best‐fitting MLPE regression models (∆AIC ≤ 2), after randomly excluding different numbers of samples (Supporting Information Figure S5).

### 2.5 Identification of putative adaptive loci

To identify genomic signatures of adaptations to local environmental conditions, we employed environmental association tests. To this end, we used our original dataset filtered by quality and depth only (as described above), but not for LD or HWE. We first ran a principal component analysis using all 19 WorldClim bioclimatic variables plus altitude and forest cover (all scaled), to select a set of orthogonal variables explaining most environmental variation across our study area. Since the first four principal components accounted for 92.08% of total variance, we selected the four variables that were most strongly correlated with these axes. The selected variables (mean temperature of coldest quarter, forest cover, temperature annual range, and precipitation of driest quarter) were then used to run latent factor mixed models (LFMMs), aiming to identify possible associations between SNPs and environmental variables, while accounting for the underlying population structure (De Kort et al., 2014; Frichot, Schoville, Bouchard, & François, 2013; Rellstab, Gugerli, Eckert, Hancock, & Holderegger, 2015). LFMMs have been used extensively and are currently one of the most commonly used environmental association analysis approaches (Ahrens et al., 2018), given they provide a good compromise between detection power and error rates, and are robust to a variety of sampling designs and underlying demographic models (Rellstab et al., 2015). LFMM were implemented in R through the LEA package, using 1,000 iterations, a burn‐in of 5,000, and five runs per environmental variable (Frichot & François, 2015). The *p*‐values were adjusted using the genomic inflation factor (*λ* = 1), and false discovery rates were set using the Benjamini–Hochberg algorithm at a rate of *q* = 0.05 (Benjamini & Hochberg, 1995). Since incorrect assumptions about underlying demographic structure can increase both type I and type II errors (Cushman & Landguth, 2010; Storfer et al., 2018), we ran LFMM using *k* ± 1 latent factors (where *k* was the optimum number of ancestral populations detected) and only considered as candidate loci those shared between all runs for each environmental variable. Full R scripts of LFMM can be found in the LEA website (http://membres-timc.imag.fr/Olivier.Francois/LEA/index.htm). In order to map adaptive genetic variability, we used the *adegenet* package (Jombart, 2008) to run a spatial principal component analysis (sPCA) on all identified candidate SNPs and interpolated the first two principal components on a grid covering our study area.

## 3 RESULTS

### 3.1 Genetic diversity and population structure

**Figure 2:**
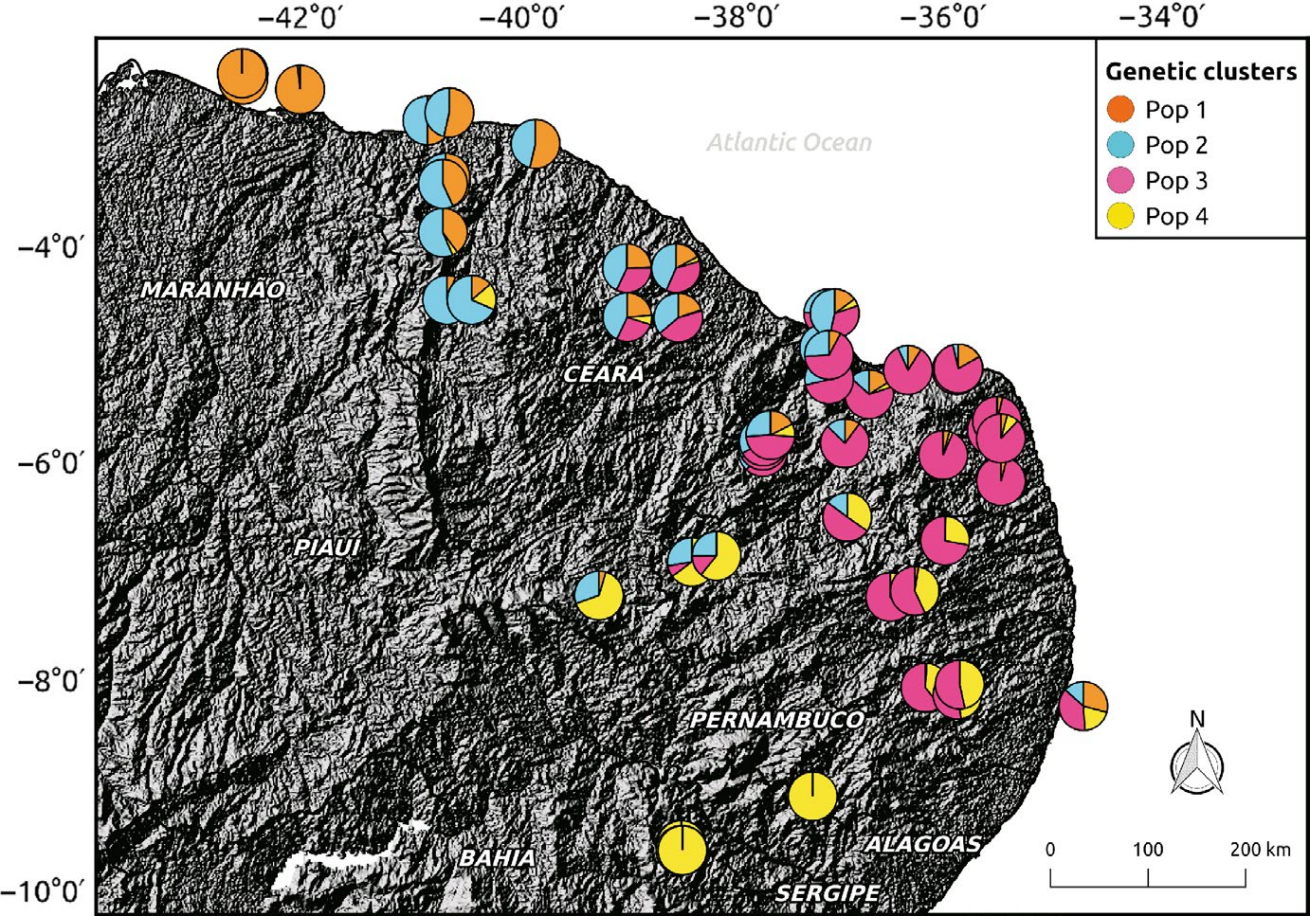
Map showing *Melipona subnitida* assignments to four genetic clusters against an elevation map (from USGS Earth Explorer). Pie charts represent ancestry coefficients determined using the LEA package

We identified 29,349 SNPs from which 3,454 loci remained after filtering for quality, depth, HWE, LD, and *F*_ST_ outlier loci. We detected four genetic clusters (*K* = 4) using two different clustering approaches (Figure 2, Supporting Information Figure S1), and excluded four samples from subsequent analyses (final sample size was 156 individuals) because they were likely introduced bees from a distant location (Supporting Information Figure S2, Table S1). Cluster assignments were generally unambiguous as ancestry coefficients for the assigned clusters were usually above 0.50 (lower 25% quantile = 0.50, median = 0.60, upper 75% quantile = 0.86). Clusters 1 and 4, located at the northern and southern extremes of the species distribution range, showed the lowest *N*_e_ and *π*. All genetic clusters showed small but significant inbreeding and values of Tajima's *D* overlapping zero (Table 1).

**Table 1:** Genetic diversity estimates for *Melipona subnitida* by genetic cluster

Genetic cluster	*N*	*N*_e_	*H*_O_	*H*_E_	*F*	*π*	Tajima's* D*
Pop 1	19	62.3/64.2	0.21/0.25	0.25/0.25	0.01/0.15	0.17/0.19	−0.09/0.36
Pop 2	32	211.9/221.0	0.22/0.22	0.23/0.23	0.03/0.05	0.21/0.22	−0.09/0.44
Pop 3	66	285.1/292.3	0.20/0.21	0.22/0.22	0.05/0.09	0.21/0.22	−0.06/0.54
Pop 4	39	107.5/109.7	0.19/0.20	0.22/0.22	0.08/0.15	0.19/0.21	−0.04/0.54

Notes

Sample sizes (*N*) are shown followed effective population size (*N*_e_), observed heterozygosity (*H*_O_), expected heterozygosity (*H*_E_), inbreeding coefficient (*F*), nucleotide diversity (*π*), and Tajima's *D*. Lower and upper 95% confidence intervals are shown for each estimate.

### 3.2 Landscape genetic analyses

**Figure 3:**
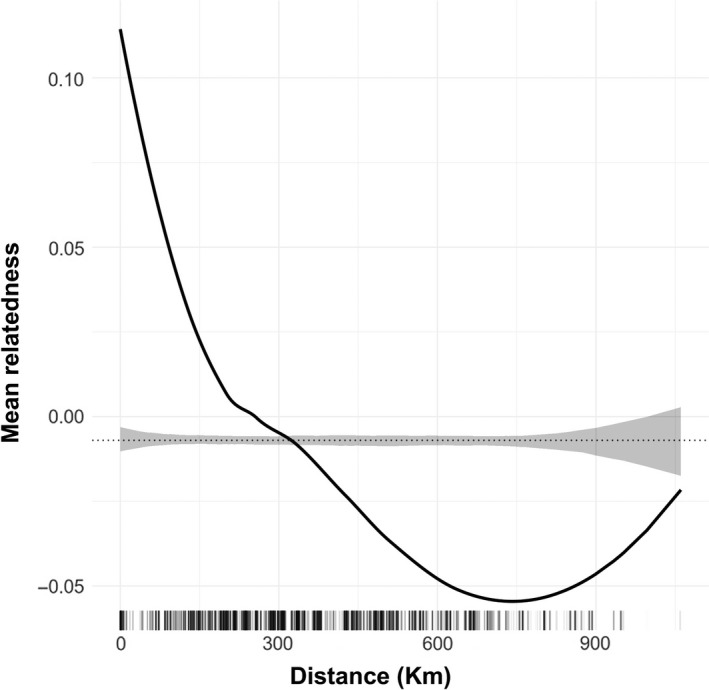
Spatial autocorrelation in genetic relatedness. The black solid line is the LOESS fit to the observed genetic relatedness, while the gray shaded regions are 95% confidence bounds around the null expectation (black dotted line). Short vertical lines at the bottom of the figure are observed pairwise distances

**Figure 4:**
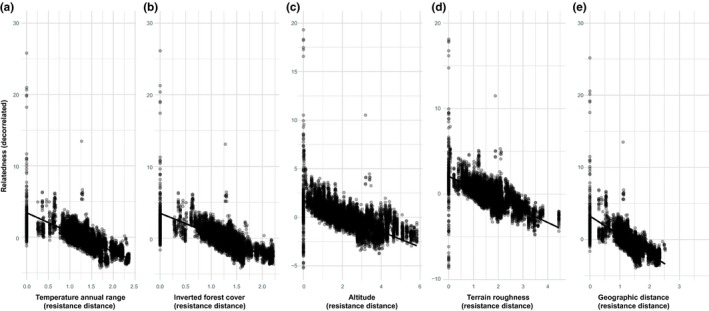
Isolation‐by‐resistance effects across the entire distribution range of *Melipona subnitida*. Plots show the relationship between genetic relatedness and temperature annual range (a), inverted forest cover (b), altitude (c), terrain roughness (d), and geographic distance (e). Although the isolation by geographic distance was not among the top models, we include it here for comparison. Relatedness is decorrelated for the MLPE correlation structure

Habitat amount was not found associated with heterozygosity or inbreeding (Table 2). We found positive spatial autocorrelation in pairwise genetic relatedness for up to 300 km, after which spatial autocorrelation became negative (Figure 3). However, IBR was better able to explain genetic relatedness patterns than IBD (Table 3). Temperature annual range (defined as the difference between maximum temperature of warmest month and minimum temperature of coldest month) was the best predictor of relatedness patterns across the full distribution range of *M. subnitida*. The second best IBR model was nearly 18 AIC units apart from the model containing temperature annual range (Table 3), suggesting that forest cover, altitude, and terrain roughness combined did not explain relatedness patterns as well as temperature fluctuations alone. All these predictors showed a significantly negative association with genetic relatedness (Table 4, Figure 4). Although temperature annual range did not show a larger variation than the other predictors used to assess IBR (Supporting Information Figure S3), it was highly correlated with forest cover (*r* = 0.94; Supporting Information Figure S4), so the effect of temperature annual range is likely confounded by forest cover to some extent. Nested MLPE models (accounting for spatial dependence) substantially improved fit and reduced evident autocorrelation. Although parameter estimates from NMLPE models had slightly larger standard errors (implying these were more conservative estimates), all effects continued to be significantly different from zero (Table 4). Our sensitivity analysis on IBR models revealed that temperature annual range, altitude, and terrain roughness were the most frequent variables included in the set of best models (Supporting Information Figure S5).

**Table 2:** Effect of habitat amount on observed heterozygosity (*H*_O_), expected heterozygosity (*H*_E_), and inbreeding coefficient (*F*)

Response variable	Correlation structure	*X* ^2^	*p*‐Value
*H*_O_	Exponential	0.24	0.63
*H*_E_	None	0.22	0.64
*F*	Exponential	0.25	0.62

Notes

Generalized least squares models contained genetic diversity metrics as response variables, percentage of habitat cover as predictor, and different correlation structures to account for spatial autocorrelation. Logit transformations were used to normalize/linearize heterozygosities. The table shows *X^2^* values and *p*‐values from likelihood ratio tests applied on best‐fitting models.

**Table 3:** Summary statistics for the top MLPE regression models

Predictors	logLik	AIC	ΔAIC	Weight	*ρ*
Temperature annual range***	22,813.91	−45,619.8	0.00	1	0.27
Inverted forest cover***, altitude***, terrain roughness*	22,807.03	−45,602.1	17.76	0	0.23
Inverted forest cover***	22,576.63	−45,145.3	474.56	0	0.25
Geographic distance	22,521.73	−45,035.5	584.37	0	0.27

Notes

All models contained interindividual genetic relatedness as response variable and the different landscape resistance distances as predictors. Log‐likelihoods are followed by the Akaike information criterion (AIC), ΔAIC, model weight, and the MLPE correlation coefficient rho (*ρ*)*.* Isolation by geographic distance was included here for comparison.

Likelihood ratio tests: *p < 0.05, **p < 0.01, * *p* < 0.001

**Table 4:** Parameter estimates for the best‐fitting MLPE regression models (ΔAIC < 20; see Table 3) and NMLPE regression models (unbiased by spatial dependence; in parentheses)

Predictors	Estimate	SE	CI
Temperature annual range	−0.14 (−0.09)	0.001 (0.003)	−0.14/−0.14 (−0.1/−0.09)
Inverted forest cover	−0.11 (−0.07)	0.002 (0.005)	−0.12/−0.11 (−0.08/−0.06)
Altitude	−0.02 (−0.01)	0.001 (0.003)	−0.02/−0.02 (−0.01/−0.001)
Terrain roughness	−0.01 (−0.02)	0.002 (0.005)	−0.01/−0.001 (−0.03/−0.01)
Geographic distance	−0.14 (−0.15)	0.001 (0.001)	−0.14/−0.14 (−0.1/−0.08)

Notes

Estimates are followed by standard errors (*SE*) and 95% confidence intervals (CI). Although the isolation by geographic distance was not among the top models, we include it here for comparison.

### 3.3 Identification of putative adaptive loci

**Figure 5:**
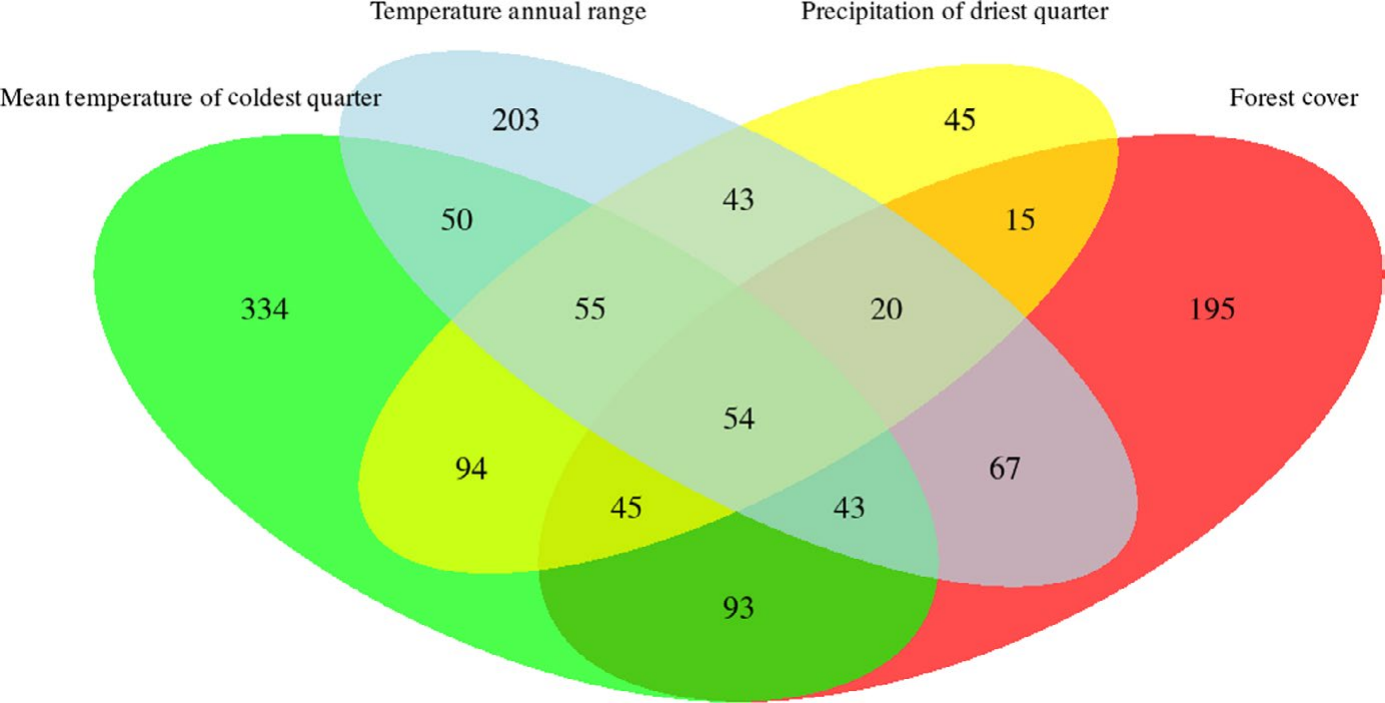
Venn diagram showing the intersection of sequences (contigs) containing candidate SNPs for *Melipona subnitida.* Putative adaptive loci were identified using environmental association tests, employing mean temperature of coldest quarter, temperature annual range, precipitation of driest quarter, and forest cover

**Figure 6:**
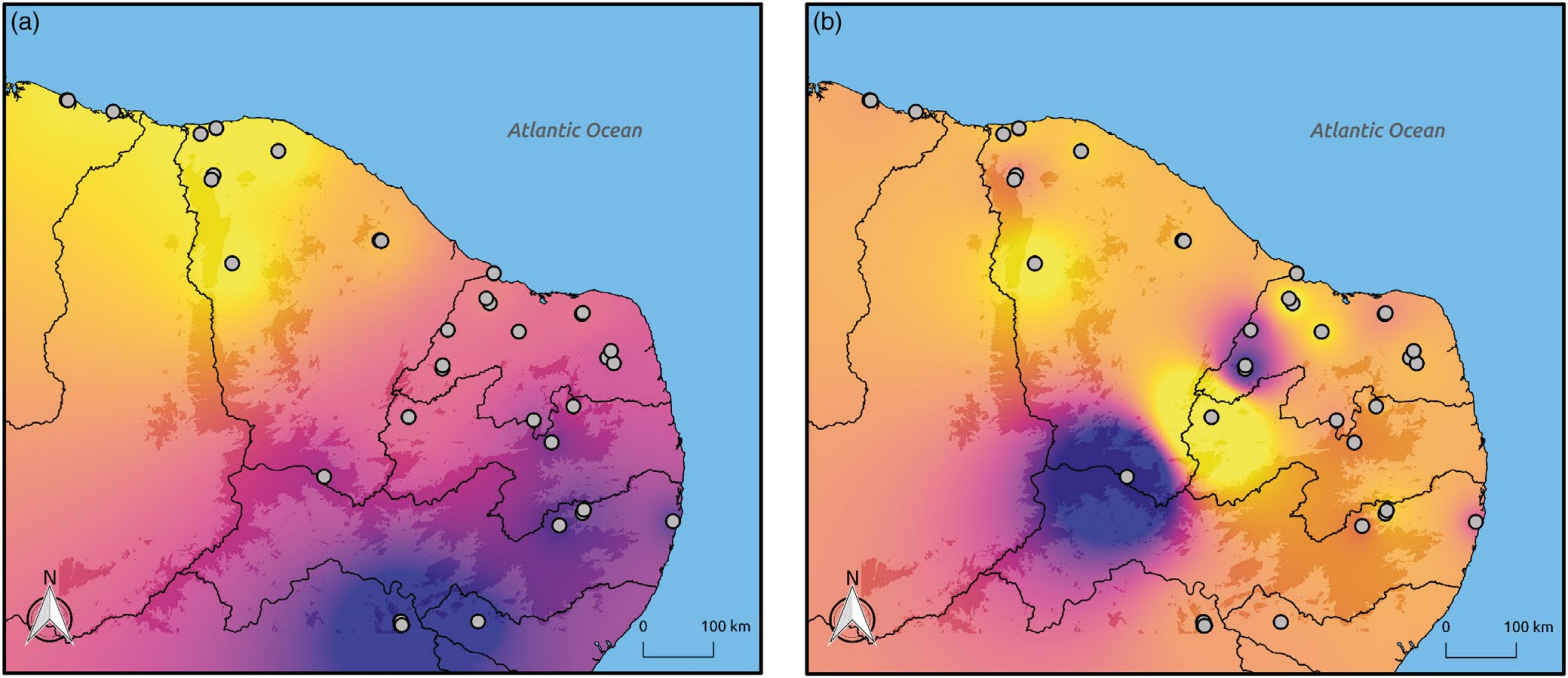
Spatial distribution of adaptive genetic variability in *Melipona subnitida*. Colors represent interpolated spatial principal components (sPCA) and suggest a latitudinal pattern associated with sPC1 (a) and an altitudinal pattern associated with sPC2 (b). Shaded areas represent elevations of at least 500 masl

About 10% of all analyzed sequences contained signatures of selection (Table 5), and most of the identified candidate loci were associated with temperature (Figure 5). The spatial distribution of adaptive genetic variability revealed latitudinal and altitudinal gradients (Figure 6).

**Table 5:** Summary of the number of adaptive signals detected employing environmental association tests. Both the number of candidate SNPs and the number of contigs (RAD tags) containing candidate SNPs are presented for each environmental predictor followed by the number of independent (nonoverlapping) detections in parentheses

Signal type	Total analyzed	Total under selection	Environmental association tests^a^			
			Mean Temp CoQ	Forest Cover	Temp AnR	Prec DrQ
SNPs	27,799	1,798	997 (444)	718 (281)	700 (261)	478 (67)
Contigs	15,924	1,356	768 (334)	532 (195)	535 (203)	371 (45)

a Environmental variables: mean temperature of coldest quarter (Mean Temp CoQ), forest cover, temperature annual range (Temp AnR), and precipitation of driest quarter (Prec DrQ).

## 4 DISCUSSION

Our study reveals a clinal change in genetic structure across the distribution range of *M. subnitida*, with four identifiable genetic clusters. Genetic diversity was not influenced by habitat amount, pairwise relatedness showed spatial autocorrelation, and isolation by resistance explained range‐wide relatedness patterns better than isolation by geographic distance. Specifically, gene flow was enhanced by low annual temperature variation, more forest cover, lower elevations, and flatter terrains. Finally, we detected genomic signatures of adaptation to temperature, precipitation, and forest cover and found latitudinal and altitudinal patterns in the spatial distribution of adaptive genetic variation.

Although previous genetic studies found intraspecific variation and signals of population structure in *M. subnitida* (Bonatti, Simões, Franco, & Francoy, 2014; Cruz et al., 2006; Silva et al., 2014), we here present the first assessment of spatial genetic structure based on thousands of independent genetic markers and performed across the full distribution range of this species. Our results reveal four genetic clusters, confirmed by two complementary methods, and extensive admixture (Figure 2). Genetic clusters located at the northernmost and southernmost extremes of the species’ distribution range (Pop 1 and Pop 4) showed lower nucleotide diversity and lower effective population size (*N*_e_) but similar heterozygosity and inbreeding as the genetic clusters located at the distribution's core (Pop 2 and Pop 3). This finding suggests that *M. subnitida* colonized these peripheral regions more recently, whereas clusters Pop 2 and Pop 3 had more time to accumulate a higher genetic variability, as stated by the central–peripheral hypothesis (Diniz‐Filho et al., 2009).

Genetic diversity was slightly higher than that reported in other genomic bee studies (Jackson et al., 2018; Romiguier et al., 2014), and in all genetic clusters we found Tajima's *D* values overlapping with zero, which indicate mutation–drift equilibrium. Additionally, we did not find a significant association between habitat amount and genetic diversity. These results suggest that genetic variation has not been influenced by habitat loss yet and that the observed levels of inbreeding are presumably related to the reproductive biology of this species (DiLeo & Wagner, 2016). Monogamy (Jaffé et al., 2014), low population densities, and sharp seasonal variations in the production of reproductive individuals (Ferreira, Blochtein, & Serrão, 2013; Roubik, 2006; Santos‐Filho, Alves, Eterovic, Imperatriz‐fonseca, & Kleinert, 2006) may result in mating between related individuals. We nevertheless caution that longer time lags may be necessary to detect an effect of habitat loss on genetic diversity (Schlaepfer, Braschler, Rusterholz, & Baur, 2018).

We detected significant spatial autocorrelation in genetic relatedness, contradicting earlier microsatellite‐based studies for other stingless bee species (Jaffé, Castilla, et al., 2016; Landaverde‐González et al., 2017). Interestingly, spatial autocorrelation was positive for up to 300 km (Figure 3), indicating that related colonies can be found across large areas and that gene flow did not erase the genetic signals left by limited colony dispersal. Above 300 km, spatial autocorrelation became negative, suggesting that population differentiation caused lower than random relatedness between individuals from different clusters (Figure 2). These results suggest it would be safe to transport colonies no further than 300 km to avoid altering the genetic composition of wild populations (Jaffé, Pope, et al., 2016).

Isolation by landscape resistance explained range‐wide relatedness patterns better than isolation by geographic distance alone. For instance, temperature fluctuations were found to be the most important factor explaining relatedness patterns in *M. subnitida,* followed by forest cover, elevation, and terrain roughness. Our IBR results hold when accounting for spatial dependence and when excluding different numbers of samples, although forest cover looses importance when more samples are excluded (Supporting Information Figure S5). Significant isolation by resistance (altitude) was only found in one other stingless bee (*Partamona helleri* Friese, 1900), out of 18 analyzed species to date (Jaffé, Pope, et al., 2016; Landaverde‐González et al., 2017), suggesting that the resolution of these microsatellite‐based studies only allowed the detection of very strong IBR effects. Our findings thus imply that fine‐scale genetic structure and IBR may be more common in this group of bees than previously acknowledged and that studies employing thousands of genetic markers and large sample sizes are needed to identify or rule out weak, but significant IBR effects.

Our results reveal that thermal stability and forest cover (which were highly correlated) are key mediators of genetic connectivity in this stingless bee species, and support earlier findings stressing out the role of elevation as a bee dispersal barrier (Jackson et al., 2018; Jaffé, Pope, et al., 2016). Although thermal range is known to have a profound influence on insect physiology (Dixon et al., 2009), ours is the first study to report an effect on dispersal behaviors. Interestingly, temperature annual range was found to be the main abiotic predictor of bee richness and diversity in the eastern Neotropics, suggesting environmental variability may have led to higher speciation (Faria & Gonçalves, 2013). Our results imply that a similar mechanism may be operating at the species level, with increased thermal variability hindering gene flow, and thus facilitating local adaptation (Allendorf et al., 2013).

Even though thermal stability was the main factor explaining gene flow patterns in *M. subnitida*, temperature annual range and forest cover were highly correlated, so we could not disentangle the relative contribution of each. Our work is nevertheless the first to reveal a significant effect of forest cover on stingless bee gene flow, a long‐standing expectation for this group of bees with restricted dispersal (Jaffé, Pope, et al., 2016). Reduced gene flow across deforested areas was only found in one other tropical organism so far (Monteiro et al., 2019), namely the army ant *Eciton burchelli* Westwood, 1842 (Pérez‐Espona, McLeod, & Franks, 2012). Interestingly, these army ants exhibit striking life history similarities with stingless bees, as queens are permanently wingless and thus show a restricted dispersal (Jaffé, Moritz, & Kraus, 2009). Forested areas thus seem important dispersal corridors for this stingless bee, which could facilitate the migration toward higher elevations predicted under climate change (Supporting Information Figure S6).

Our environmental association tests can be considered robust to deviations from the underlying demographic structure, as candidate loci were intersected across different *k*‐values. Additionally, LFMM were calibrated based on the distribution of adjusted *p*‐values, so the incidence of false discovery rates was low (François et al., 2016). Interestingly, we found latitudinal and altitudinal gradients in the distribution of adaptive genetic variation. While the former gradient was also found in the honeybee *A. mellifera* (Chávez‐Galarza et al., 2013; Henriques et al., 2018), and is probably related to climatic conditions, the altitudinal gradient suggests different adaptations to current highland and lowland areas. Our findings thus reveal the presence of locally adapted bees, which should be preserved to maintain evolutionary potential (Hoffmann & Sgrò, 2011; Sgrò, Lowe, & Hoffmann, 2011). While lowland populations of *M. subnitida* are expected to shift to higher elevations by 2050 (Supporting Information Figure S6), current highland populations are at risk, since highlands will have no climate analogs in the future (Colwell, Brehm, Cardelús, Gilman, & Longino, 2008; Giannini et al., 2017). Conservation actions should thus prioritize the protection of current highland populations while improving the connectivity between highlands and lowlands, preserving or restoring foothill and mountain forests (Giannini et al., 2017).

Taken together, our findings shed important light on the life history of *M. subnitida* and highlight the role of regions with large thermal fluctuations, deforested areas, and mountain ranges as dispersal barriers. Moreover, our work unravels previously unknown patterns of local adaptation in these bees. This knowledge could help guide future conservation actions such as avoiding the transportation of colonies beyond 300 km, preserving highland and lowland populations separately, and conserving or restoring foothill and mountain forests. Considering the high biological and economic importance of this native pollinator, such conservation efforts will be easily offset if honey production and pollination services are maintained in the future.

## DATA ARCHIVING STATEMENT

Genotype and sequence data have been deposited in FigShare: https://doi.org/10.6084/m9.figshare.7863416.v1

## CONFLICT OF INTEREST

None declared.
