# Supplementary material for: Landscape genomics to the rescue of a tropical bee threatened by habitat loss and climate change
Source: Evol Appl. 2019 Apr 10;12(6):1164–77. doi: 10.1111/eva.12794 (PMC6597871; doi:10.1111/eva.12794)
Supplement: Supplementary file 1 [file EVA-12-1164-s001.pdf]

**Supporting Information**

**Figures**

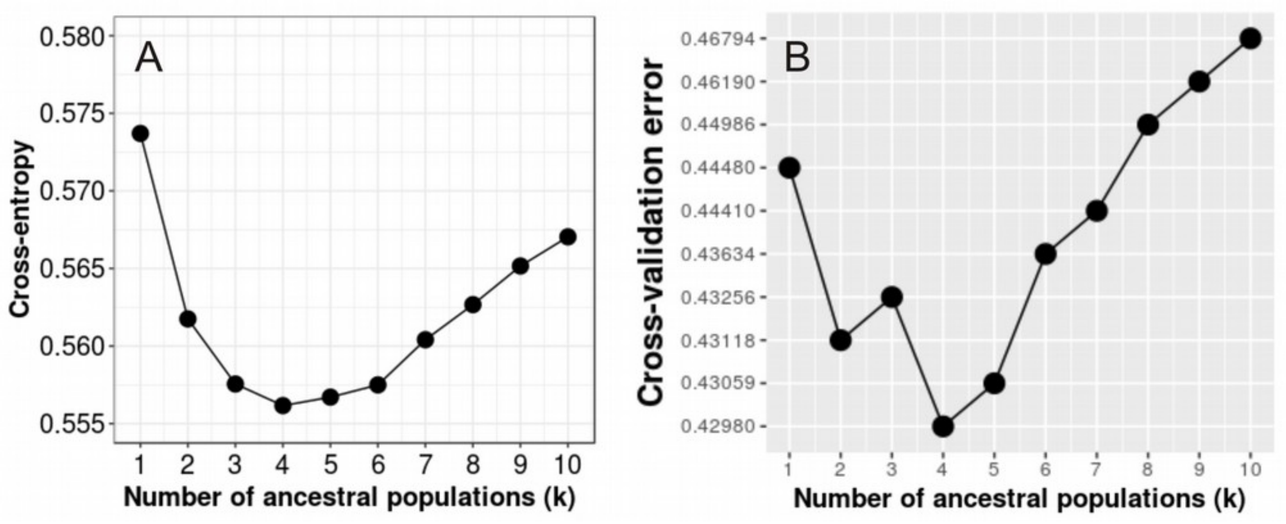


**Figure S1:** Plots showing the optimal number of genetic clusters. Optimal k choice is based on mean ± sd cross-entropy (LEA, A) and cross-validation errors (Admixture, B).

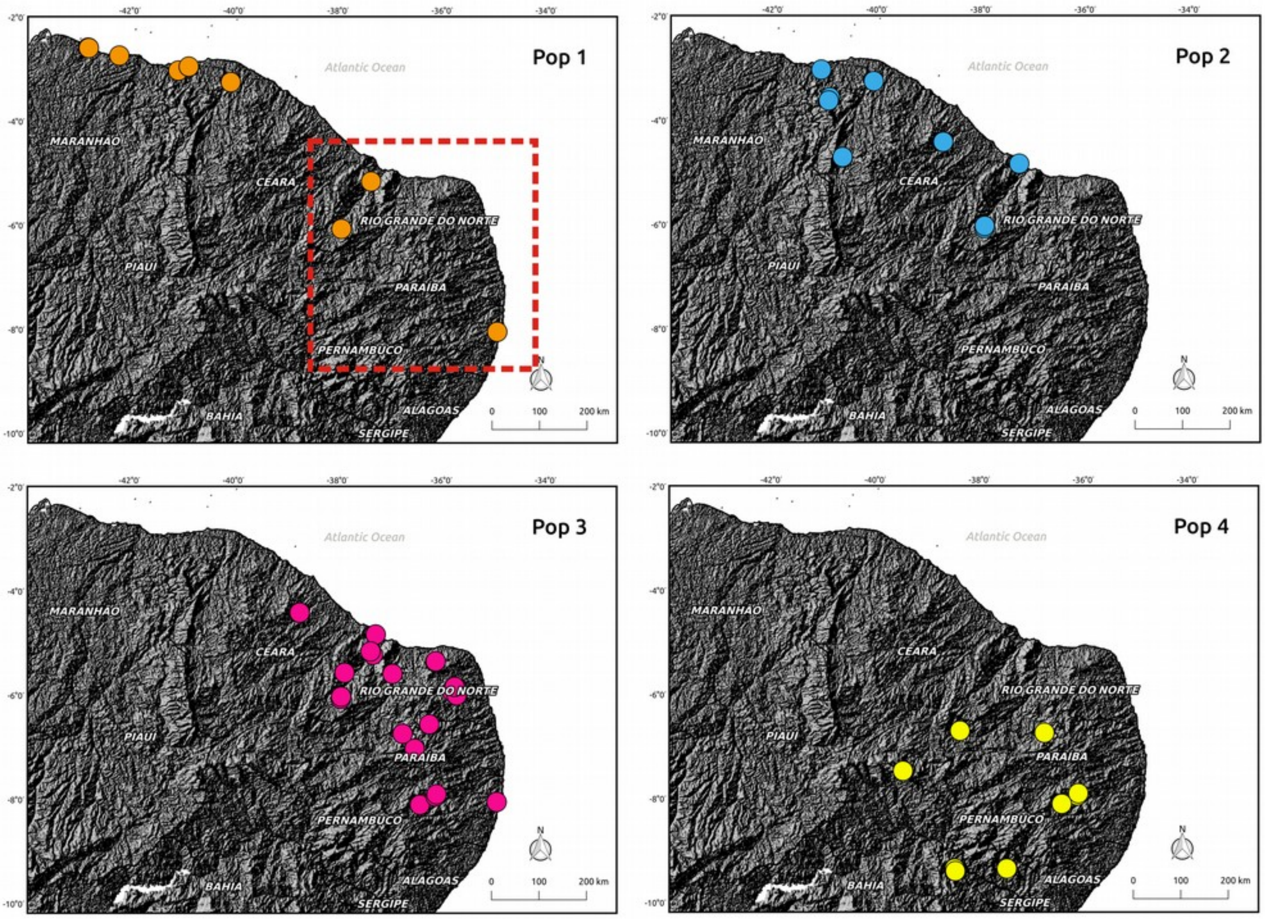


**Figure S2:** Maps showing *Melipona subnitida* assignments to four genetic clusters against an elevation map (from USGS Earth Explorer). The red square shows the excluded samples from Pop 1 (orange cluster), given they were likely introduced bees.

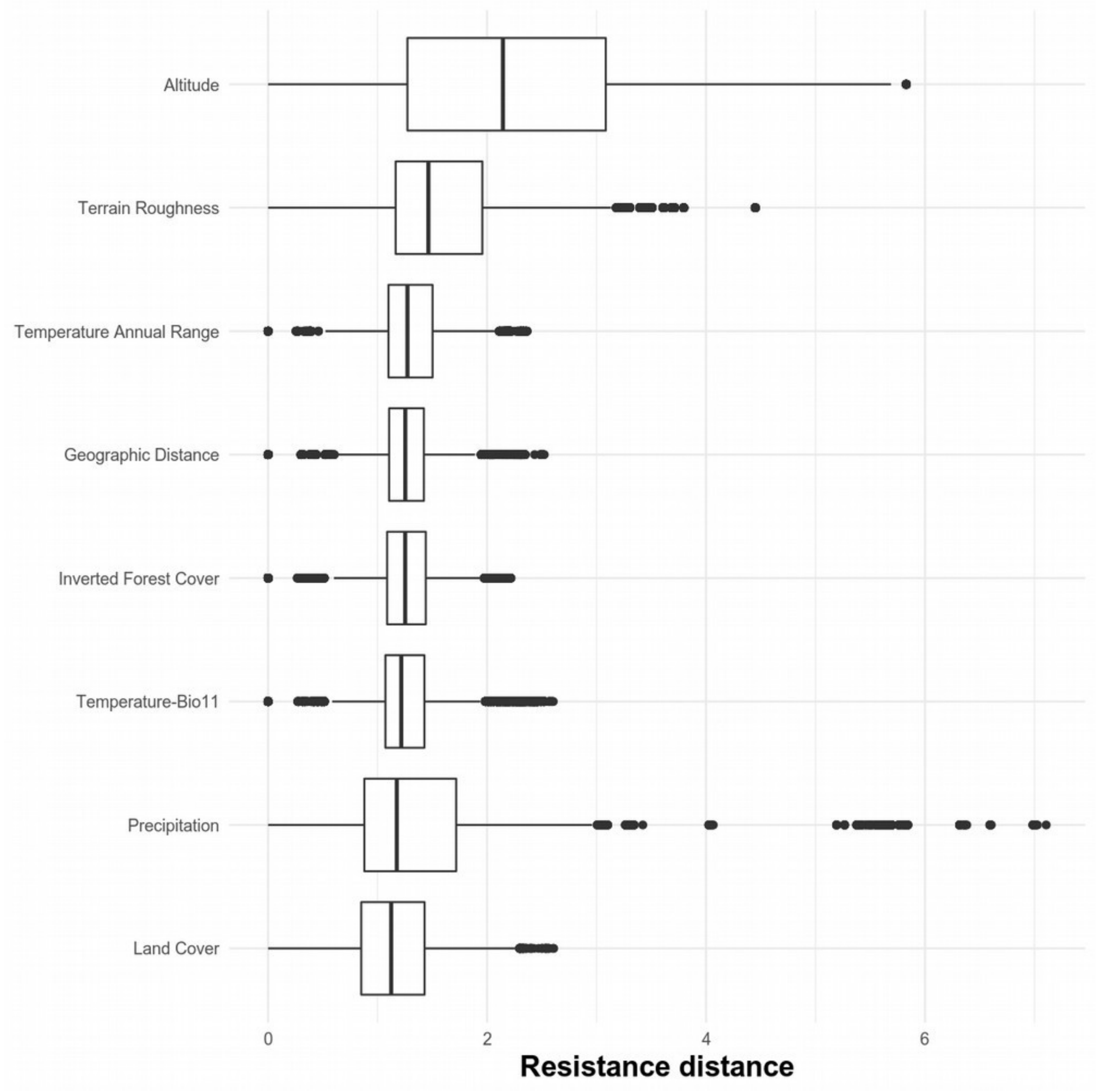


**Figure S3:** Variable ranges for all pairwise resistance distances used to run MLPE regression models.

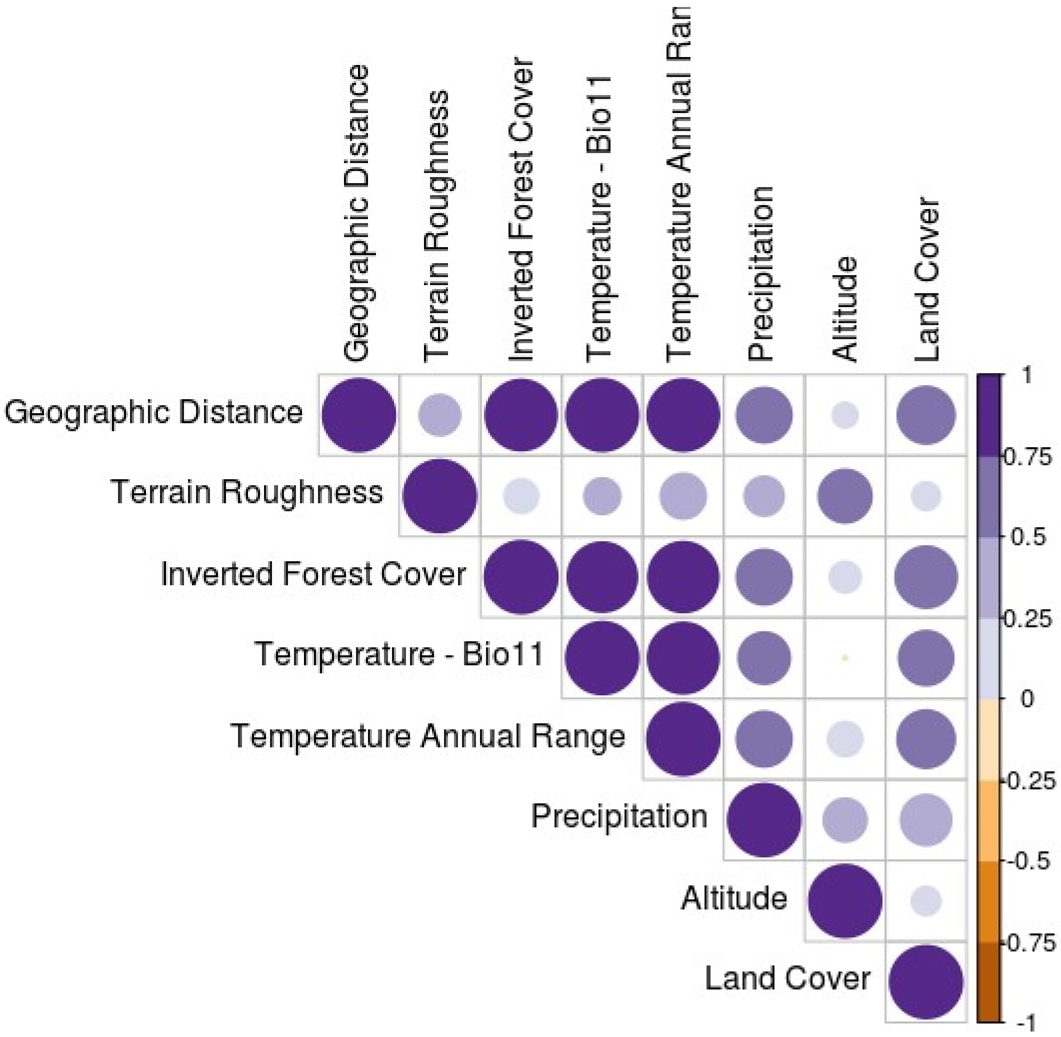


**Figure S4:** Correlogram showing the correlation between all resistance distances included as predictors in MLPE regression models. Pearson's correlation coefficients (*r*) are depicted by colors and circle sizes.

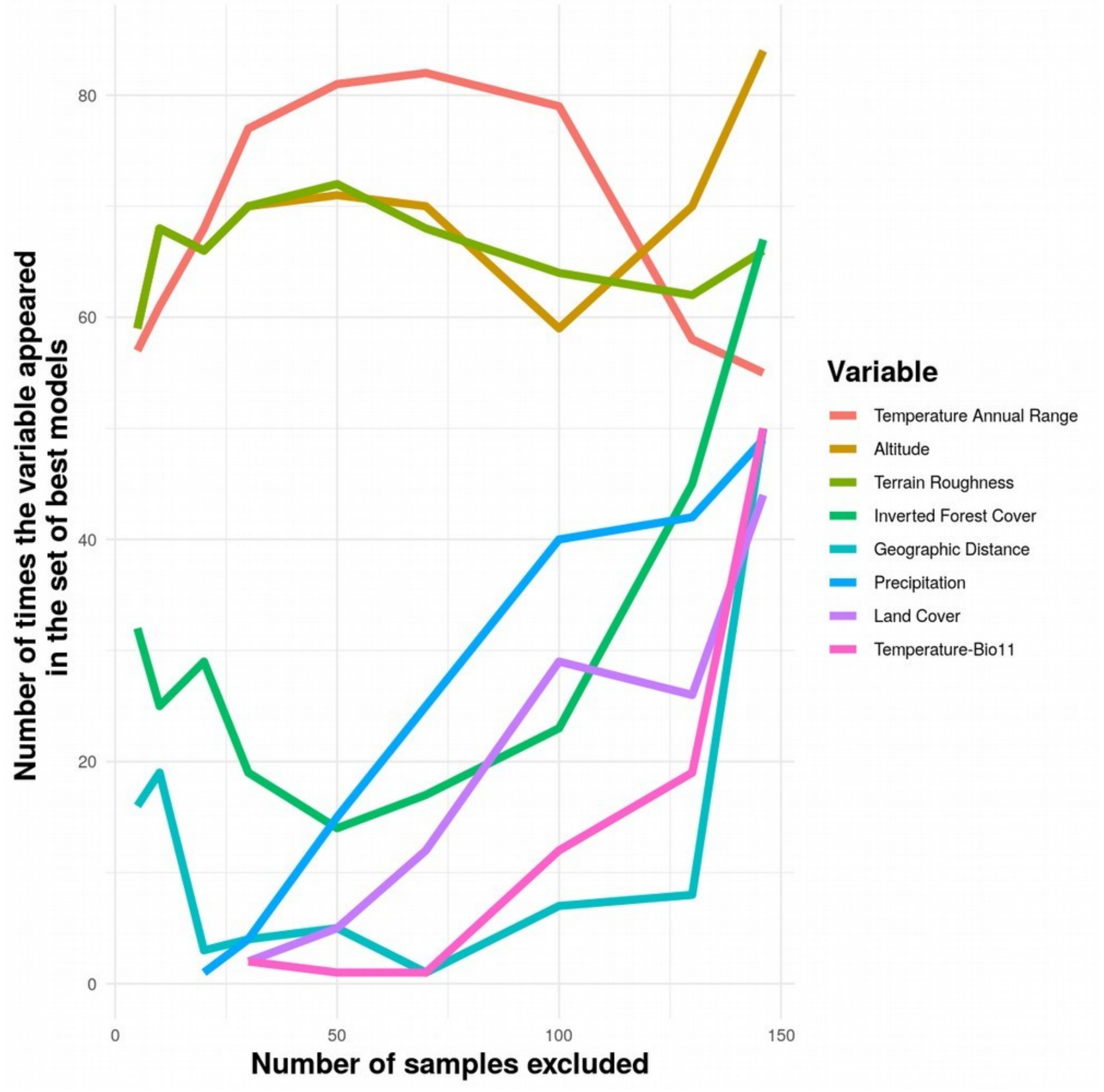


**Figure S5:** Sensitivity analyses showing the number of times predictor variables were included in the set of best-fitting MLPE regression models (ΔAIC ≤ 2), after randomly excluding different numbers of samples. One hundred data subsets were generated and one hundred independent model selection protocols were performed for each treatment (number of excluded samples: 5, 10, 20, 30, 50, 70, 100, 130, and 146).

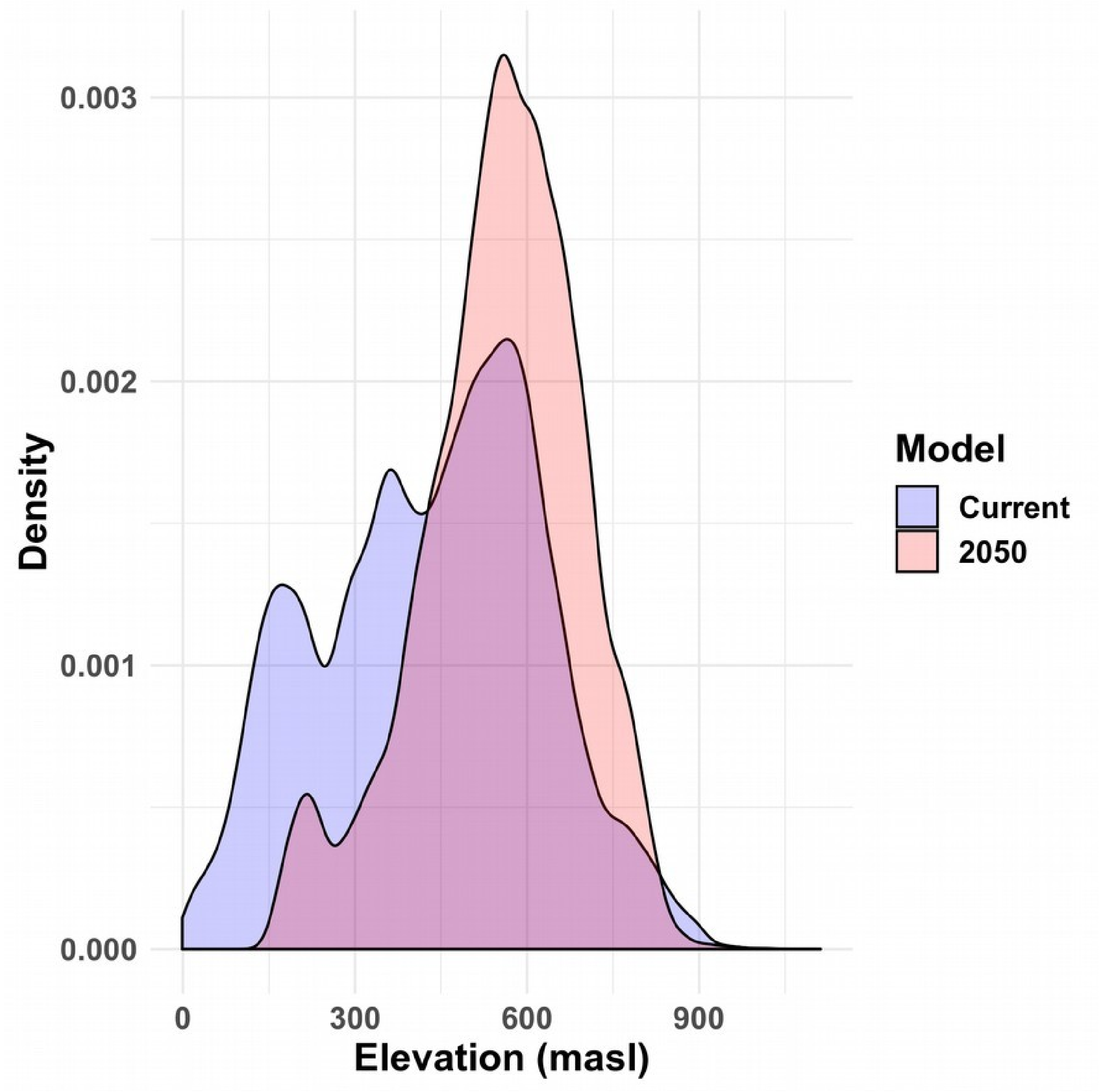


**Figure S6:** Density plots showing the current and future (2050) distribution of elevations where *M. subnitida* is most likely to occur across Northeastern Brazil (probability of occurrence ≥ 50%), according to the species distribution models (SDM) by Giannini et al. (2017). SDM were cropped to the extent of our sampling locations and a Wilcoxon test was employed to compare both distributions ($W = 6.7_x10^8$, $p < 0.001$). The maximum altitude were samples were collected in our study was 872.3 masl.

**Tables**

**Table S1:** Information on the collected *Melipona subnitida* samples, including sample ID, location name, State, and geographic coordinates (in decimal degrees).

| *ID* | *Location name* | *State* | *Latitude* | *Longitude* |
|---|---|---|---|---|
| J03* | Martins | RN | -6.06348300352692 | -37.9369529988616 |
| J04 | Martins | RN | -6.06348300352692 | -37.9369529988616 |
| J06 | Martins | RN | -6.06348300352692 | -37.9369529988616 |
| J07 | Martins | RN | -6.06348300352692 | -37.9369529988616 |
| J08 | Martins | RN | -6.06348300352692 | -37.9369529988616 |
| J09 | Martins | RN | -6.06348300352692 | -37.9369529988616 |
| J10 | Martins | RN | -6.06348300352692 | -37.9369529988616 |
| J12 | Pé da Serra de Martins | RN | -6.02193902246654 | -37.9327550064772 |
| J13 | Pé da Serra de Martins | RN | -6.02193902246654 | -37.9327550064772 |
| J14 | Pé da Serra de Martins | RN | -6.02193902246654 | -37.9327550064772 |
| J15 | Pé da Serra de Martins | RN | -6.02193902246654 | -37.9327550064772 |
| J16 | Pé da Serra de Martins | RN | -6.02193902246654 | -37.9327550064772 |
| J17 | Açu | RN | -5.5826364364475 | -36.9447094202041 |
| J18 | Açu | RN | -5.5826364364475 | -36.9447094202041 |
| J19 | Açu | RN | -5.5826364364475 | -36.9447094202041 |
| J20 | Açu | RN | -5.5826364364475 | -36.9447094202041 |
| J21 | Açu | RN | -5.5826364364475 | -36.9447094202041 |
| J24 | Aroeiras, Jandaira | RN | -5.3541820 | -36.1265490 |
| J25 | Aroeiras, Jandaira | RN | -5.3541820 | -36.1265490 |
| J26 | Aroeiras, Jandaira | RN | -5.3541820 | -36.1265490 |
| J27 | Aroeiras, Jandaira | RN | -5.3541820 | -36.1265490 |
| J28 | Aroeiras, Jandaira | RN | -5.3541820 | -36.1265490 |
| J30 | Jandaíra | RN | -5.3543428145349 | -36.1266305577009 |

| | | | | |
|---|---|---|---|---|
| | | | | |
| J31 | Jandaíra | RN | -5.34169116988778 | -36.1143727786839 |
| J32 | Jandaíra | RN | -5.34169116988778 | -36.1143727786839 |
| J33 | Jandaíra | RN | -5.34169116988778 | -36.1143727786839 |
| J34 | Jandaíra | RN | -5.34169116988778 | -36.1143727786839 |
| J35 | Jandaíra | RN | -5.34169116988778 | -36.1143727786839 |
| J36 | Aroeiras, Jandaira | RN | -5.3541820 | -36.1265490 |
| J40 | S. Paulo Potengi | RN | -5.9219220 | -35.7980690 |
| J41 | S. Paulo Potengi | RN | -5.9219220 | -35.7980690 |
| J42 | S. Paulo Potengi | RN | -5.9219220 | -35.7980690 |
| J43 | S. Paulo Potengi | RN | -5.9219220 | -35.7980690 |
| J44 | S. Paulo Potengi | RN | -5.9219220 | -35.7980690 |
| J45 | Sta. Maria | RN | -5.83328191190958 | -35.75014882721 |
| J46 | Sta. Maria | RN | -5.83328191190958 | -35.75014882721 |
| J47 | Sta. Maria | RN | -5.83328191190958 | -35.75014882721 |
| J48 | Sta. Maria | RN | -5.83328191190958 | -35.75014882721 |
| J49 | Eloy de Souza | RN | -5.99348857998848 | -35.7126027625054 |
| J50 | Eloy de Souza | RN | -5.99348857998848 | -35.7126027625054 |
| J51 | Eloy de Souza | RN | -5.99348857998848 | -35.7126027625054 |
| J54 | Jardim do Seridó | RN | -6.73099103383719 | -36.7511167936027 |
| J55 | Jardim do Seridó | RN | -6.73099103383719 | -36.7511167936027 |
| J56 | Jardim do Seridó | RN | -6.73315666615963 | -36.7505053337663 |
| J57 | Jardim do Seridó | RN | -6.73315666615963 | -36.7505053337663 |
| J58 | Joazerinho | RN | -7.02022436074912 | -36.5198833029717 |
| J59 | Joazerinho | RN | -7.02022436074912 | -36.5198833029717 |
| J60 | Joazerinho | RN | -7.02022436074912 | -36.5198833029717 |
| J61 | Joazerinho | RN | -7.02022436074912 | -36.5198833029717 |

| | | | | |
|---|---|---|---|---|
| J62 | Joazerinho | RN | -7.02022436074912 | -36.5198833029717 |
| J65 | Aurora da Serra, Apodi | RN | -5.56177144870162 | -37.8667800407856 |
| J66 | Aurora da Serra, Apodi | RN | -5.56177144870162 | -37.8667800407856 |
| J67 | Aurora da Serra, Apodi | RN | -5.56177144870162 | -37.8667800407856 |
| J68 | Aurora da Serra, Apodi | RN | -5.56177144870162 | -37.8667800407856 |
| J70 | Mossoró | RN | -5.21579196676611 | -37.3215300310403 |
| J71 | Mossoró | RN | -5.21579196676611 | -37.3215300310403 |
| J72 | Mossoró | RN | -5.21579196676611 | -37.3215300310403 |
| J73 | Barrinha, Icapuí | CE | -4.82721707783639 | -37.268912801519 |
| J74 | Barrinha, Icapuí | CE | -4.82721707783639 | -37.268912801519 |
| J75 | Barrinha, Icapuí | CE | -4.82721707783639 | -37.268912801519 |
| J76 | Barrinha, Icapuí | CE | -4.82721707783639 | -37.268912801519 |
| J78 | Arapá, Tianguá | CE | -3.6133560 | -40.9317650 |
| J79 | Arapá, Tianguá | CE | -3.6133560 | -40.9317650 |
| J80 | Arapá, Tianguá | CE | -3.6133560 | -40.9317650 |
| J81 | Arapá, Tianguá | CE | -3.6133560 | -40.9317650 |
| J82 | Sitio Letreiro, Tianguá | CE | -3.5538220 | -40.9067260 |
| J83 | Sitio Letreiro, Tianguá | CE | -3.5538220 | -40.9067260 |
| J84 | Sitio Letreiro, Tianguá | CE | -3.5538220 | -40.9067260 |
| J85 | Sitio Letreiro, Tianguá | CE | -3.5538220 | -40.9067260 |
| J86 | Sitio Letreiro, Tianguá | CE | -3.5538220 | -40.9067260 |
| J87 | Sitio Letreiro, Tianguá | CE | -3.5538220 | -40.9067260 |
| J88 | Lagoa Sto. Antonio, Aurora, Ararendá | CE | -4.698096960783 | -40.6634870264679 |
| J89 | Lagoa Sto. Antonio, Aurora, Ararendá | CE | -4.698096960783 | -40.6634870264679 |
| J90 | Lagoa Sto. Antonio, Aurora, Ararendá | CE | -4.698096960783 | -40.6634870264679 |
| J91 | Lagoa Sto. Antonio, Aurora, Ararendá | CE | -4.698096960783 | -40.6634870264679 |

| | | | | |
|---|---|---|---|---|
| | | | | |
| J92 | Lagoa Sto. Antonio, Aurora, Ararendá | CE | -4.698096960783 | -40.6634870264679 |
| J93 | Lagoa Sto. Antonio, Aurora, Ararendá | CE | -4.698096960783 | -40.6634870264679 |
| J94 | Lagoa Sto. Antonio, Aurora, Ararendá | CE | -4.698096960783 | -40.6634870264679 |
| J95 | Camocim | CE | -3.020333 | -41.073278 |
| J98 | Camocim | CE | -2.946417 | -40.871417 |
| J99 | Camocim | CE | -3.020333 | -41.073278 |
| J100 | Camocim | CE | -3.020389 | -41.072944 |
| J101 | Camocim | CE | -2.946417 | -40.871417 |
| J102 | Camocim | CE | -3.020333 | -41.073278 |
| J104 | Camocim | CE | -2.946417 | -40.871417 |
| J105 | Morrinhos | CE | -3.241444 | -40.064528 |
| J107 | Morrinhos | CE | -3.241444 | -40.064528 |
| J112 | Camocim | CE | -2.942528 | -40.871417 |
| J116 | Morrinhos | CE | -3.241444 | -40.064528 |
| J117 | Morrinhos | CE | -3.241444 | -40.064528 |
| J118 | Camaragibe | PE | -8.0433 | -34.9438 |
| J120 | Taquaritinga do Norte | PE | -7.895722 | -36.099611 |
| J121 | Taquaritinga do Norte | PE | -7.895722 | -36.099611 |
| J123 | Taquaritinga do Norte | PE | -7.895722 | -36.099611 |
| J124 | Taquaritinga do Norte | PE | -7.895722 | -36.099611 |
| J128 | Taquaritinga do Norte | PE | -7.93725 | -36.11825 |
| J131 | Taquaritinga do Norte | PE | -7.93725 | -36.11825 |
| J132* | Camaragibe | PE | -8.04329599253833 | -34.9438010249286 |
| J133* | Camaragibe | PE | -8.04329599253833 | -34.9438010249286 |
| J157 | Aracoiaba | CE | -4.40326795913279 | -38.7486160174012 |
| J158 | Aracoiaba | CE | -4.40326795913279 | -38.7486160174012 |

| | | | | |
|---|---|---|---|---|
| J161 | Aracoiaba | CE | -4.40095799043774 | -38.7514920160174 |
| J162 | Aracoiaba | CE | -4.40095799043774 | -38.7514920160174 |
| J164 | Aracoiaba | CE | -4.40095799043774 | -38.7514920160174 |
| J165 | Aracoiaba | CE | -4.40095799043774 | -38.7514920160174 |
| J171 | Aracoiaba | CE | -4.40717300400137 | -38.7294359598308 |
| J172 | Aracoiaba | CE | -4.40717300400137 | -38.7294359598308 |
| J173 | Aracoiaba | CE | -4.40717300400137 | -38.7294359598308 |
| J178 | Aracoiaba | CE | -4.40228501334786 | -38.7326169759035 |
| J179 | Aracoiaba | CE | -4.40228501334786 | -38.7326169759035 |
| J180 | Aracoiaba | CE | -4.40228501334786 | -38.7326169759035 |
| J220 | São João do Rio do Peixe | PB | -6.69141403399407 | -38.3740939758718 |
| J221 | São João do Rio do Peixe | PB | -6.69141403399407 | -38.3740939758718 |
| J222 | São João do Rio do Peixe | PB | -6.69141403399407 | -38.3740939758718 |
| J223 | São João do Rio do Peixe | PB | -6.69141403399407 | -38.3740939758718 |
| J224 | São João do Rio do Peixe | PB | -6.69141403399407 | -38.3740939758718 |
| J225 | São João do Rio do Peixe | PB | -6.69141403399407 | -38.3740939758718 |
| J226 | São João do Rio do Peixe | PB | -6.69141403399407 | -38.3740939758718 |
| J227 | São João do Rio do Peixe | PB | -6.69141403399407 | -38.3740939758718 |
| J228 | São João do Rio do Peixe | PB | -6.69141403399407 | -38.3740939758718 |
| J230 | São João do Rio do Peixe | PB | -6.69141403399407 | -38.3740939758718 |
| J231 | São João do Rio do Peixe | PB | -6.69141403399407 | -38.3740939758718 |
| J232 | São João do Rio do Peixe | PB | -6.69141403399407 | -38.3740939758718 |
| J244 | Ararendá | CE | -4.69809402711689 | -40.6635489687323 |
| J245 | Ararendá | CE | -4.69809402711689 | -40.6635489687323 |
| J246 | Brejo da Madre de Deus | PE | -8.09712499380111 | -36.41880299896 |
| J247 | Brejo da Madre de Deus | PE | -8.09712499380111 | -36.41880299896 |

| | | | | |
|---|---|---|---|---|
| J250 | Brejo da Madre de Deus | PE | -8.09712499380111 | -36.41880299896 |
| J251 | Mourelandia | PE | -7.464116960763930 | -39.470182964578200 |
| J252 | Mourelandia | PE | -7.464116960763930 | -39.470182964578200 |
| J255 | Mourelandia | PE | -7.464116960763930 | -39.470182964578200 |
| J256 | Mourelandia | PE | -7.464116960763930 | -39.470182964578200 |
| J257 | Mourelandia | PE | -7.464116960763930 | -39.470182964578200 |
| J259 | Mourelandia | PE | -7.464116960763930 | -39.470182964578200 |
| J261 | Mourelandia | PE | -7.464116960763930 | -39.470182964578200 |
| J262* | Tibau | RN | -5.152516979724160 | -37.367801992222600 |
| J263 | Tibau | RN | -5.152516979724160 | -37.367801992222600 |
| J264 | PNLM | MA | -2.584867 | -42.796867 |
| J265 | PNLM | MA | -2.585267 | -42.793383 |
| J266 | PNLM | MA | -2.585233 | -42.793383 |
| J269 | PNLM | MA | -2.58175 | -42.795367 |
| J270 | PNLM | MA | -2.580633 | -42.79435 |
| J272 | PNLM | MA | -2.583283 | -42.797883 |
| J274 | PNLM | MA | -2.5793333 | -42.8062667 |
| J275 | PNLM | MA | -2.5795833 | -42.8103667 |
| J276 | Tutóia | MA | -2.730692 | -42.206304 |
| J277 | Brejo do Brugo | BA | -9.3428333 | -38.4743889 |
| J278 | Brejo do Brugo | BA | -9.3413889 | -38.4828611 |
| J279 | Brejo do Brugo | BA | -9.3413889 | -38.4828611 |
| J282 | Brejo do Brugo | BA | -9.3426667 | -38.47375 |
| J283 | Brejo do Brugo | BA | -9.3426667 | -37.47375 |
| J284 | Brejo do Brugo | BA | -9.3425556 | -38.4739167 |
| J289 | Brejo do Brugo | BA | -9.3891111 | -38.4563333 |

| | | | | |
|---|---|---|---|---|
| | | | | |
| J290 | Brejo do Brugo | BA | -9.3891389 | -38.4563056 |
| J292 | Brejo do Brugo | BA | -9.3911111 | -38.4636111 |
| J293 | Brejo do Brugo | BA | -9.3911111 | -38.4636111 |
| J294 | Brejo do Brugo | BA | -9.3911111 | -38.4636111 |
| J296 | Brejo do Brugo | BA | -9.391 | -38.4786944 |
| J297 | Picuí | PB | -6.551574972 | -36.23790904 |
| J298 | Picuí | PB | -6.551574972 | -36.23790904 |

* Beekeepers reported previous introductions of bee colonies in these locations, and genetic clustering analyses revealed that these samples were attributed to distant populations. We therefore excluded these samples from subsequent analyses.
